# Quantitative Evaluation of Gait Changes Using APDM Inertial Sensors After the External Lumbar Drain in Patients With Idiopathic Normal Pressure Hydrocephalus

**DOI:** 10.3389/fneur.2021.635044

**Published:** 2021-07-08

**Authors:** Mengmeng He, Zhenyu Qi, Yunxiang Shao, Hui Yao, Xuewen Zhang, Yang Zhang, Yu Shi, Qinzhi E, Chengming Liu, Hongwei Hu, Jiangang Liu, Xiaoou Sun, Zhong Wang, Yulun Huang

**Affiliations:** ^1^Department of Neurosurgery, The First Affiliated Hospital of Soochow University, Suzhou, China; ^2^Department of Neurosurgery, Dushu Lake Hospital Affiliated of Soochow University, Suzhou, China

**Keywords:** gait impairment, idiopathic normal pressure hydrocephalus, APDM, Inertial sensors, quantitative analysis

## Abstract

**Objectives:** Gait and balance disturbances are common symptoms of idiopathic normal pressure hydrocephalus (iNPH). This study aimed to quantitatively evaluate gait and balance parameters after external lumbar drainage (ELD) using APDM inertial sensors.

**Methods:** Two-minute walkway tests were performed in 36 patients with suspected iNPH and 20 healthy controls. A total of 36 patients underwent ELD. According to clinical outcomes, 20 patients were defined as responders, and the other 16 as non-responders. The gait parameters were documented, and the corresponding differences between responders and non-responders were calculated.

**Results:** When compared with healthy controls, patients with suspected iNPH exhibited decreased cadence, reduced gait speed, a higher percentage of double support, decreased elevation at mid-swing, reduced foot strike angle, shorter stride length, difficulty in turning, and impaired balance functions. After the ELD, all these manifestations, except elevation at mid-swing and balance functions, were significantly improved in responders. The change of *Z*-score absolute value in the six parameters, except for foot strike angle, was >1. No significant improvement was observed in non-responders.

**Conclusion:** APDM inertial sensors are useful for the quantitative assessment of gait impairment in patients with iNPH, which may be a valuable tool for identifying candidates that are suitable for shunting operations.

## Introduction

Idiopathic normal pressure hydrocephalus (iNPH), characterized by dilated cerebral ventricles and normal cerebrospinal fluid (CSF) pressure, was first described by Hakim and Adams (1). Clinically, iNPH manifests as gait disturbance, cognitive deficiency, and urinary incontinence. In recent years, with the rapid aging of society in general, the prevalence of iNPH is continuously increasing. However, iNPH is usually underdiagnosed as some of the existing symptoms may be considered as aging-related degeneration (2). Additionally, iNPH is often misdiagnosed as Parkinson disease, Alzheimer disease, vascular dementia, or musculoskeletal diseases because they share common characteristics, such as gait abnormalities and dementia. Unfortunately, the delay in diagnosis may hinder therapeutic efficacy (3). Therefore, it is crucial to develop an objective and quantitative method for identifying iNPH.

Prior to surgical treatment, temporary CSF drainage, including the CSF tap test (CSFTT) and external lumbar drainage (ELD), is the first choice for identifying patients suitable for shunting. Patients who have experienced symptomatic improvement after temporary CSF drainage may benefit from shunting surgery (4). The CSFTT has a high specificity even up to 100% in some studies. However, it is insensitive; i.e., it has only limited negative predictive value (5). It has been reported that the sensitivity of ELD is higher than that of the CSFTT (6, 7). Therefore, in the present study, we chose to utilize the ELD trial rather than the CSFTT to assess the suitability of iNPH patients for shunting.

Walking patterns are significantly associated with individual health status and provide early clinical evidence for a potential gait disorder (8, 9). Gait and balance impairments therefore represent the main manifestations of iNPH, which are usually described as small-stepped, magnet gait or broad-based gait (10, 11). Gait assessment is an important tool for screening surgical patients who are iNPH positive after temporary CSF drainage. Commonly used gait assessments in clinical and research-based iNPH work include subjective evaluations, functional ambulation profiles, and objective metrics, such as those obtained from employing a stopwatch, electromyography, an electronic walkway, optical motion capture (OMC) systems, and wearable inertial sensors (12, 13). In general, subjective evaluations depend highly on personal experience and are not quantitative, and measurements using mechanized instruments may eliminate, such a manual bias. The Ambulatory Parkinson's Disease Monitoring (APDM) inertial sensor (Opals and Mobility Lab) is a new wearable system that may facilitate objective motion analyses (14–16). The portable body-worn opal sensors include three-axis accelerometers, gyroscopes, and a magnetometer, which can automatically analyze gait and balance information, and generate a detailed report. The validity and reliability of the APDM have been proven in various patient populations. Herein, we used APDM to quantitatively analyze gait and balance parameters in iNPH patients undergoing ELD.

## Materials and Methods

### Participants

This retrospective study enrolled 40 patients from our hospital between August 2018 and July 2020. The diagnosis of iNPH was made according to the Tokyo guideline (11). The study was approved by the ethics committee of The First Affiliated Hospital of Soochow University, and all participants provided written informed consent prior to undergoing any CSF drainage test. The inclusion criteria were as follows: (1) age ≥60 years; (2) gait disturbances with or without cognitive dysfunctions or urinary incontinence; (3) no causative neurological or non-neurological disorders or apparent preceding disorders that may cause hydrocephalus; (4) dilated ventricles with an Evans ratio of >0.3 (the ratio of the width of the frontal horns of the lateral ventricles to the maximal internal diameter of the skull on computed tomography/magnetic resonance imaging (MRI scan); and (5) disproportionately enlarged subarachnoid space hydrocephalus observed on MRI. Exclusion criteria included (1) unable to ambulate for ≥2 min; (2) previous history of head trauma, intracerebral hemorrhage, meningitis, or other diseases that may cause secondary hydrocephalus; or (3) symptoms explained by other causes, such as spinal stenosis and fracture. Additionally, gait analysis with APDM was performed in 20 healthy adults (≥60 years) with normal neurological functions, and no active neurological, systemic, or psychiatric disorders.

### Gait Analysis

**Figure 1:**
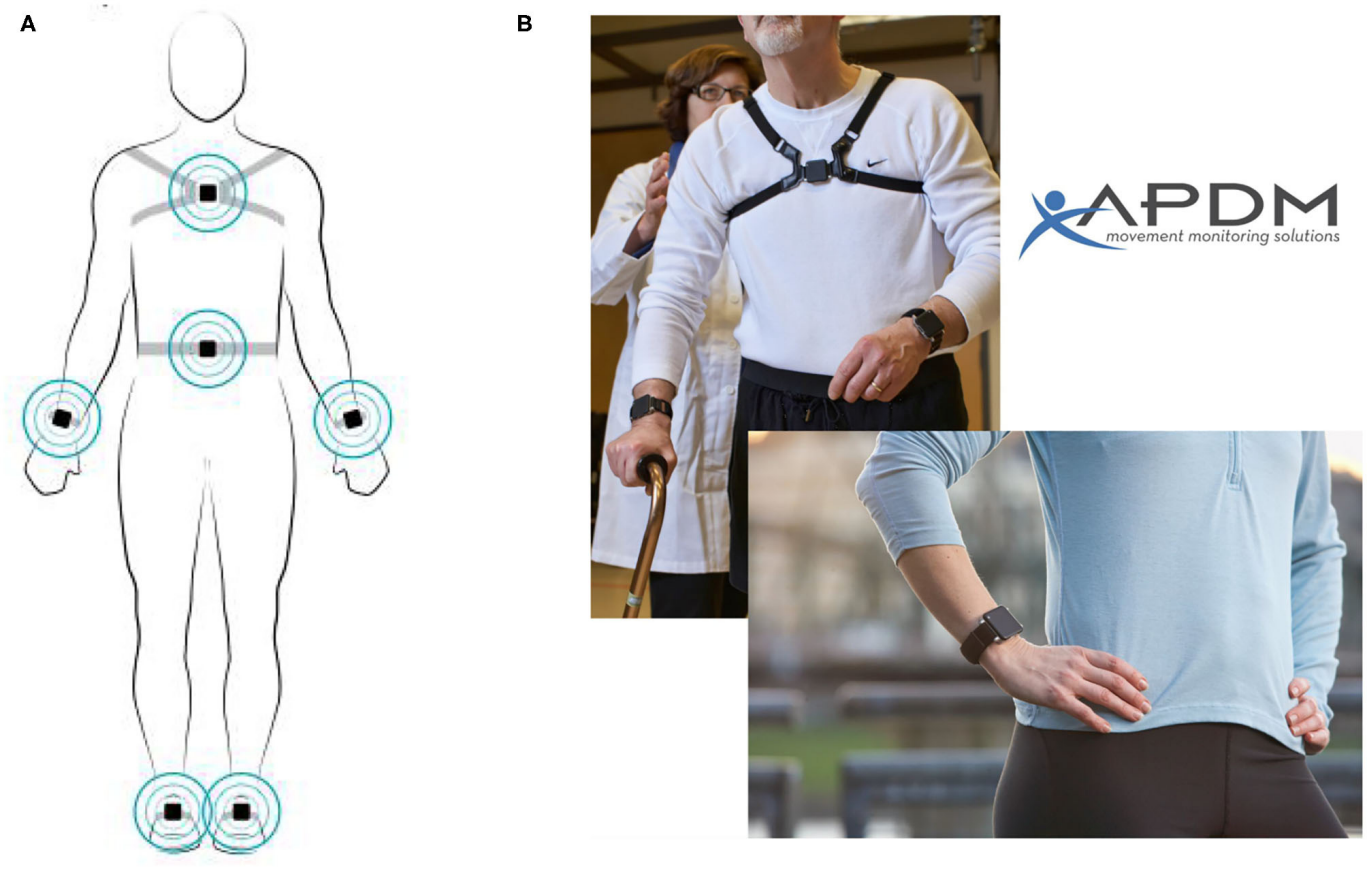
Sensors on the body. Reproduced with permission from APDM Inc. **(A)** Location of Opal sensors. **(B)** Example of sensors attached to the sternum and bilateral wrists.

Gait analysis was performed using a wireless APDM Movement Monitoring inertial sensor system (APDM Inc., USA). Inertial sensors, attached by elastic Velcro straps, were placed on the bilateral wrists and feet, as well as on the sternum and the fifth lumbar vertebrae (Figure 1). Gait and balance parameters were collected from prescribed tasks.

Two-minute walkway tests were performed with the APDM at 1 h before and 4 h after the ELD. Participants were asked to walk forward on a straight 7-m walkway at a comfortable pace for 2 min. To avoid falling during the test, a physical therapist walked with the subject. The test was repeated three times for all participants, and the best performance was selected for statistical analyses.

The Grading Scale for iNPH (INPHGS) was used to evaluate the severity of iNPH-related symptoms (i.e., cognitive impairment, gait disturbance, and urinary disturbance). The Global Rating of Change (GRC) scale was used to assess individual self-perception. This is a visual scale with ratings ranging from −5 to +5, in which −5 represents completely worse, 0 indicates no change, and +5 indicates complete improvement.

### ELD Surgery

ELD surgery was performed by a neurosurgeon 1 h after gait examination. The CSF drainage velocity was set at 10 ml/h for 48 h. After the ELD, the patients were asked to adapt to ambulation for 4 h. Then, the gait analysis with APDM was repeated, and the GRC assessment was completed.

#### Definition of Response

Three neurosurgeons and one geriatrician with 10–25 years of experience evaluated the clinical response. All radiological profiles, gait examinations, and self-reports of patients were comprehensively analyzed. The patients were categorized into responders and non-responders. Surgical treatment was recommended for the responders.

#### Data Processing and Statistical Analyses

The gait parameters were quantitatively evaluated and compared among the responders, non-responders, and normal controls. Comparisons between responders and non-responders were performed to determine the significance of changes after ELD. The IBM SPSS Statistics software for Windows (version 20.0.0) was used for statistical analyses. Categorical variables were compared using a χ^2^ test, and continuous variables were compared using a Student *t* test. Spearman correlation analysis was used to calculate correlation coefficients between GRC scores and gait parameters. The statistical significance threshold was set at *P* < 0.05.

## Results

### Participants

A total of 40 patients were invited to participate in this study. Two patients refused participation, and two patients were excluded because of concomitant diseases. Eventually, 36 patients completed the ELD, and gait examinations. The demographic and clinical symptoms of responders and non-responders are summarized in Table 1. There were no differences in age, gender, presence of symptoms, or INPHGS scores between responders and non-responders (all *P* > 0.05).

**Table 1:** Demographic and clinical characteristics of participants.

**Characteristics**	**Responders** ** (*n* = 20)**	**Non-responders** ** (*n* = 16)**	***p* Value**
Age (years)	73.5 (5.8)	69.6 (5.9)	0.06
Gender (male/female)	15/5	12/4	1.00
Symptom duration (month)	12.8 (10.6)	14.8 (13.0)	0.62
Percentage of the presence of symptoms (gait/ cognition/incontinence)	100%/70%/50%	94%/56%/25%	0.58
Grading Scale for iNPH	4.4 (2.0)	4.1 (1.7)	0.61
Patients with diabetes mellitus	6	4	0.59
Patients with hypertension	9	8	0.99

### Gait Analysis

Gait parameters included cadence, gait speed, percentage of double support, foot strike angle, lateral step variability, stride length, coronal range of lumber motion, and steps in turning. All these parameters in responders and non-responders were impaired when compared to normal controls (all *P* < 0.05), whereas no significant differences were noted between responders and non-responders (all *P* > 0.05). The mean values of gait parameters before ELD are summarized in Table 2.

**Table 2:** Mean values of gait parameters before ELD.

**Parameter**	**Responders** ** (*n* = 20)**	**Non-responders** ** (*n* = 16)**	**Control group ** **(*n* = 20)**	***p* value responders vs. non-responders** ** (95% CI)**	***p* value controls vs. responders** ** (95% CI)**	***p* value controls vs. non-responders** ** (95% CI)**
Cadence (steps/min)	100.16 (17.14)	103.93 (14.37)	109.37 (7.22)	0.493 (−14.81, 7.27)	0.038 (−17.87, −0.54)	0.037 (−12.96, −0.41)
Gait speed (m/s)	0.46 (0.18)	0.58 (0.29)	1.08 (0.82)	0.180 (−0.29, 0.06)	0.000 (−0.71, −0.52)	0.000 (−0.66, −0.34)
Percentage of double support (%)	32.55 (7.85)	30.20 (7.26)	18.68 (2.46)	0.369 (−2.90, 7.60)	0.000 (10.02, 17.73)	0.000 (7.47, 15.58)
Elevation at mid-swing (cm)	1.42 (0.75)	1.34 (0.80)	2.23 (0.68)	0.723 (−0.40, 0.57)	0.000 (−1.20, −0.40)	0.000 (−1.35, −0.43)
Foot strike angle (°)	6.72 (6.00)	9.78 (8.53)	19.30 (5.15)	0.233 (−8.22, 2.10)	0.000 (−16.05, −9.11)	0.000 (−14.51, −4.53)
Toe-out angle (°)	11.24 (10.07)	15.74 (8.50)	8.07 (6.18)	0.073 (−9.44, 0.45)	0.021 (0.71, 7.87)	0.000 (3.74, 11.59)
Lateral step variability (cm)	2.68 (1.50)	3.07 (1.40)	4.06 (0.91)	0.441 (−1.38, 0.61)	0.001 (−2.17, −0.58)	0.014 (−1.77, −0.22)
Stride length (m)	0.56 (0.22)	0.64 (0.34)	1.17 (1.10)	0.411 (−0.28, 0.12)	0.000 (−0.72, −0.49)	0.000 (−0.70, −0.34)
Coronal range of lumber motion (°)	3.85 (1.54)	4.44 (1.53)	7.31 (1.44)	0.534 (−1.39, 0.73)	0.000 (−4.42, −2.53)	0.000 (−4.17, −2.11)
Steps in turning (number)	5.83 (1.11)	5.51 (1.01)	3.39 (0.48)	0.371 (−0.40, 1.05)	0.000 (1.87, 3.00)	0.000 (1.54, 2.69)

We further compared gait parameters after ELD in the responder and non-responder groups. The mean values of gait parameters before and after ELD, as well as *Z*-score values of improvement after ELD, are presented in Table 3. Improvements in cadence (*P* = 0.000), gait speed (*P* = 0.000), percentage of double support (*P* = 0.000), foot strike angle (*P* = 0.002), stride length (*P* = 0.000), and steps in turning (*P* = 0.000) were significantly greater in responders compared to non-responders. The increase of *Z*-score absolute values in cadence, gait velocity, percentage of double support, stride length, and steps in turning was >1 in responders. However, there was no significant improvement in gait parameters in non-responders after discharge. Therefore, these patients with a negative response to ELD were not recommended for shunting surgery.

**Table 3:** Mean values of gait parameters before and after ELD and *Z*-score values of improvement after ELD.

**Gait parameter**	**Responders**				**Non-responders**			
	**Pre-CFSTT mean (SD)**	**Post-CFSTT mean (SD)**	***Z* score value of increasing**	***p* value before** ** vs. after ELD** ** (95% CI)**	**Pre-CFSTT mean (SD)**	**Post-CFSTT mean (SD)**	***Z* score value of increasing**	***p* value before** ** vs. after ELD** ** (95% CI)**
Cadence (steps/min)	100.16 (17.14)	107.78 (12.10)	1.67	0.000 (−11.38, −3.85)	103.93 (14.37)	104.10 (15.83)	0.03	0.905 (−3.07, 2.74)
Gait speed (m/s)	0.46 (0.18)	0.58 (0.21)	1.34	0.000 (−0.15, −0.09)	0.58 (0.29)	0.55 (0.30)	−0.24	0.082 (−0.00, 0.06)
Percentage of double support (%)	32.55 (7.85)	28.48 (7.33)	−2.30	0.000 (3.09, 5.07)	30.20 (7.26)	30.96 (7.45)	0.34	0.142 (−1.81, 0.29)
Elevation at mid-swing (cm)	1.42 (0.75)	1.47 (0.82)	0.18	0.582 (−0.25, 0.15)	1.34 (0.80)	1.23 (0.73)	−0.22	0.102 (−0.02, 0.23)
Foot strike angle (°)	6.72 (6.00)	8.16 (6.85)	0.56	0.002 (−2.26, −0.62)	9.78 (8.53)	8.91 (8.41)	−0.27	0.878 (−0.62, 0.71)
Toe-out angle (°)	11.24 (10.07)	11.01 (10.45)	−0.02	0.755 (−1.26, 1.71)	15.74 (10.28)	14.77 (10.97)	−0.25	0.535 (−2.29, 4.24)
Lateral step variability (cm)	2.68 (1.50)	3.18 (1.91)	1.199	0.060 (−1.01, 0.02)	3.07 (1.40)	2.91 (1.63)	−0.611	0.355 (−0.19, 0.50)
Stride length (cm)	0.56 (0.22)	0.66 (0.24)	1.23	0.000 (−0.13, −0.07)	0.64 (0.34)	0.64 (0.33)	0.01	0.974 (−0.08, 0.08)
Coronal range of lumber motion (°)	3.85 (1.54)	4.05 (1.36)	0.09	0.255 (−0.58, 0.16)	4.44 (1.53)	4.26 (1.55)	−0.06	0.796 (−0.42, 0.54)
Steps in turning (number)	5.83 (1.11)	4.96 (0.94)	−1.38	0.000 (−94.83, −72.74)	5.51 (1.01)	5.17 (0.99)	−0.40	0.742 (−0.50, 0.69)

### GRC Scores

The correlation scores between gait parameter changes and GRC scores after ELD are summarized in Table 4. There were statistically significant correlations between GRC scores and all gait parameters except foot strike angle. GRC scores in responders were significantly correlated with gait speed (*r* = 0.471), percentage of double support (*r* = −0.459), and steps in turning (*r* = −0.444). We speculate that there may be subtle relationships between the brain control system of these three parameters and the self-perception system of the patient. There was no significant correlation between gait parameter changes and GRC scores in non-responders.

**Table 4:** Correlations between gait parameter change values and global rating of change scores.

**Gait parameters**	**Overall**	**Responders**	**Non-responders**
Cadence	*r* = 0.516, *p* < 0.01	*r* = 0.262, *p* = 0.265	*r* = 0.144, *p* = 0.595
Gait speed	*r* = 0.737, *p* < 0.01	*r* = 0.471, *p* = 0.036	*r* = 0.171, *p* = 0.527
Percentage of double support	*r* = −0.761, *p* < 0.01	*r* = −0.459, *p* = 0.042	*r* = −0.039, *p* = 0.887
Foot strike angle	*r* = 0.274, *p* = 0.106	*r* = 0.016, *p* = 0.948	*r* = −0.295, *p* = 0.268
Stride length	*r* = 0.668, *p* < 0.01	*r* = 0.419, *p* = 0.066	*r* = 0.223, *p* = 0.407
Steps in turning	*r* = −0.527, *p* < 0.01	*r* = −0.444, *p* = 0.050	*r* = −0.195, *p* = 0.468

## Discussion

Previous studies have reported that the gait features used in determining the diagnosis of iNPH are not specific for identifying individuals who responded to tap tests (17). However, some quantitative studies have shown contrary results (4, 18–20). Our study provides evidence that gait and balance parameters are useful for quantifying changes after ELD in patients with iNPH. Compared with age- and gender-matched healthy controls, the gait of patients with iNPH was characterized by a decreased cadence, a lower gait speed, a higher percentage of double support, a decreased elevation at mid-swing, a decreased foot strike angle, a shorter stride length, difficulty in turning, and impaired balance functions. After the ELD, significant differences were apparent between responders and non-responders in six gait parameters, including the cadence, gait velocity, percentage of double support, foot strike angle, stride length, and steps in turning. Additionally, the absolute values of *Z* score in cadence, gait velocity, percentage of double support, stride length, and steps in turning were >1 in responders, indicating a positive response to the ELD. Non-responders showed negative responses to ELD, and therefore, we quantitatively evaluated the changes of gait parameters before and after the ELD.

Lower gait velocity represents the primary manifestations of iNPH, which may be remarkably improved after the CSF removal test. As observed by Stolze et al. (21), the improvement of gait velocity is attributed to the increased stride length rather than the cadence. However, our findings revealed that stride length and cadence were consistently increased after the ELD. Therefore, correlation between three parameters needs to be further verified.

In recent years, research on the percentage of double support has continuously increased. Winter et al. (22) studied changes in the biomechanical walking pattern in healthy elderly individuals and found that double-limb support is a stabilizing factor during a normal gait cycle. Panciani et al. (18) observed a significant reduction in the duration of double support, which was obviously improved after the CSFTT. In the current study, we observed an increased percentage of double support to stabilize inefficient gait control in responders before the ELD, and the duration of double support was improved after the ELD.

The foot strike angle is strongly associated with elevation at mid-swing within the gait pattern. The reduced elevation at mid-swing is attributed to the insufficient dorsal extension of the forefoot at the late swing phase, which also results in a reduced foot strike angle. Foot strike angle gradually declines with aging (16), and a more gentle foot strike is a strategy to deal with falling risks on slippery ground (23). Our study found that the foot strike angle was decreased to keep balance in patients with iNPH and was significantly improved after the ELD. However, no improvement in elevation at mid-swing was noted.

Turning difficulty is the main manifestation of gait disorders in some patients with iNPH. Approximately 30% of patients in this study had difficulty turning and also exhibited freezing and hesitant gaits. Bovonsunthonchai et al. (24) found that the number of steps in turning was sensitive to detecting motor improvement after the tap test. Souza et al. (25) also described complete turning (requiring three or more steps for turning 180°) as the most affected feature after the CSFTT (25), which was supported by our study. However, steps for turning 360°were not significantly improved in a prospective study (26). We speculate these inconsistent results may be due to inevitable biases in subjective assessments, and quantitative measurements for assessing the efficacy of ELD are warranted.

Improvement in gait and balance parameters after temporary CSF removal is considered the most important indicator for undergoing shunting surgery. Gait velocity is the best gait parameter for predicting the outcome after shunting. However, few studies focused on quantitative measures of balance-related parameters. As is well known, falls caused by balance disorders are the second leading cause of accidental or unintentional injury deaths worldwide (27). The balance-related performance, such as the Romberg eyes open and tandem stance eyes open tests, will take longer to show the benefits of continuous CSF drainage (26). A study conducted by Japanese scholars indicated that falls and imbalance were strongly associated with gait variability, which was an independent fall-related factor in patients with iNPH (28). Our study showed that balance-related parameters, including the variability of toe-out angle, lateral step variability, and coronal range of lumber motion, were significantly altered in iNPH patients. The lateral step variability in patients with iNPH was lower than that in controls, which may be due to their impaired ability to manipulate lateral balance by integrative sensorimotor control (29). However, the variability of toe-out angle and coronal range of lumber motion in patients with iNPH were greater than healthy controls, which represent manifestations of gait instability. We speculate that the changes in balance-related parameters after temporary CSF removal may provide evidence for walking improvement. However, these results are contrary to a previous study that measured footmarks of 10 iNPH patients using the traditional paper-and-pencil method and found that the variability of foot rotation angle was reduced after CSF drainage (21). This inconsistency may be attributed to the small sample size and semi quantitative measurements used in the previous study.

The patients in the current work seemed to be able to accurately perceive gait and balance changes after the ELD trial. There were significant correlations between five improved gait parameters and GRC scores. Gait speed, percentage of double support, and steps in turning were significantly correlated with GRC scores in responders. These results indicate that there may be a close relationship between the brain control system of these three parameters and the self-perception of the patients. Therefore, the definitive correlations between the self-perception of patients and gait control systems need to be further explored to confirm such a relationship.

The quantitative analyses of gait and balance parameters in the current study showed consistent results with previous studies using the OMC system (30, 31). The OMC system is the gold standard for testing gait; however, this system requires expensive equipment and a specific room where the assessment can be conducted. Wearable inertial sensors have a small size, low weight, and high sensitivity and, in addition, are mobile, low cost, and easy to operate. This gait testing modality only needs a standard walkway and does not require the presence of an experienced neurologist, and thus, it can be conveniently used in laboratory, clinic, and even home setting. An increasing amount of research has indicated that portable systems based on body sensors are promising methods for gait analyses (13). The validity and reliability of the APDM Movement Monitoring inertial sensor system have been proven in various studies. Morris et al. (32) explored the association between cognition and comprehensive gait and static balance in patients with Parkinson disease using APDM inertial sensors. Purcell et al. (33) used the APDM inertial sensors to identify the effects of dual-task cognitive interference and environmental challenges on balance in Huntington disease. In the current study, the APDM inertial sensors were proven to be effective for identifying the changes of gait and balance parameters in patients with iNPH, and even subtle changes after temporal CSF removal.

### Study Limitations

First, the sample size in the current study was small. Second, we mainly focused on the changes in gait after the ELD, whereas the changes in cognition and urinary incontinence, as well as changes in neuroimaging markers, were not evaluated. Third, the responders and non-responders were defined by medical specialists, and there has been no uniform definition of these two categories. In short, more research is needed in the future to draw a definitive conclusion on how responder and non-responder iNPH patients react to ELD.

## Conclusion

The APDM inertial sensor system is a useful tool for the quantitative assessment of gait impairment in patients with iNPH and may be valuable for identifying candidates that are suitable for shunting operations.

## Data Availability Statement

The original contributions presented in the study are included in the article/supplementary material, further inquiries can be directed to the corresponding author/s.

## Ethics Statement

The studies involving human participants were reviewed and approved by the ethics committee of The First Affiliated Hospital of Soochow University. The patients/participants provided their written informed consent to participate in this study.

## Author Contributions

MH: drafting/revision of the manuscript for content and including medical writing for content. ZQ: drafting/revision of the manuscript for content, including medical writing for content, and analysis or interpretation of data. YS, HY, and YZ: major role in the acquisition of data. YS, QE, CL, HH, JL, and XS: study concept or design. ZW: study concept or design and analysis or interpretation of data. YH: drafting/revision of the manuscript for content, including medical writing for content, study concept or design, and analysis or interpretation of data. All authors have read and approved the final manuscript.

## Conflict of Interest

The authors declare that the research was conducted in the absence of any commercial or financial relationships that could be construed as a potential conflict of interest.
